# Development and Validation of an Analytical Method Based on HPLC-ELSD for the Simultaneous Determination of Rosmarinic Acid, Carnosol, Carnosic Acid, Oleanolic Acid and Ursolic Acid in Rosemary

**DOI:** 10.3390/molecules24020323

**Published:** 2019-01-17

**Authors:** Penghui Li, Ailing Liu, Yinhua Li, Bin Yuan, Wenjun Xiao, Zhonghua Liu, Sheng Zhang, Haiyan Lin

**Affiliations:** 1 National Research Center of Engineering Technology for Utilization of Functional Ingredients from Botanicals, Hunan Agricultural University, Changsha 410128, China; lpflstt111@163.com (P.L.); liyinhua1012@126.com (Y.L.); xiaowenjun88@sina.com (W.X.); larkin-liu@163.com (Z.L.); 2 Collaborative Innovation Centre of Utilization of Functional Ingredients from Botanicals, Hunan Agricultural University, Changsha 410128, China; 3 College of Bioscience and Biotechnology, Hunan Agricultural University, Changsha 410128, China; liuailing@hunau.edu.cn (A.L.); yuanbinxueshu0516@163.com (B.Y.)

**Keywords:** rosemary, rosmarinic acid, carnosol, carnosic acid, oleanolic acid, ursolic acid, HPLC-ELSD

## Abstract

The safety, efficacy and stability of natural antioxidants have been the focus of research in the food industry, with the aim of rapidly analyzing and controlling the quality of rosemary and its extracts, a novel analytical method involving high-performance liquid chromatography with evaporative light scattering detection (HPLC-ELSD) was developed for the simultaneous determination of rosmarinic acid, carnosol, carnosic acid, oleanolic acid and ursolic acid in rosemary. Chromatographic separation was conducted with gradient elution mode by using a Zorbax SB-C18 column (4.6 mm × 250 mm, 5 μm) with mobile phases of methanol and 0.6% acetic acid. The drift tube temperature of ELSD was 70 °C, and the pressure of nebulizer nitrogen gas was 40 Psi. The method developed has high sensitivity (with limits of detection from 1.3 to 8.6 μg/mL), acceptable linearity over the tested concentrations (with correlation coefficients from 0.991 to 0.999), good repeatability (with intra- and inter-day CV less than 3.1% for all analytes) and satisfactory accuracy (with recovery between 95.5% and 100.8%). The method has been demonstrated as a powerful tool for the functional ingredients analysis and quality control of rosemary and its extracts in a cost- and time-effective manner.

## 1. Introduction

**Figure 1:**
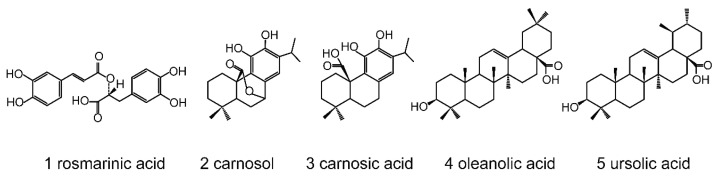
Structures of rosemary phytochemicals. **1**, rosmarinic acid (C18H16O8, WM 360.32); **2**, carnosol (C20H26O4, WM 330.42); **3**, carnosic acid (C20H28O4, WM 332.44); **4**, oleanolic acid (C30H48O3, WM 456.71); **5**, ursolic acid (C30H48O3, WM 456.71).

Rosemary (*Rosmarinus officinalis* L.) is an aromatic evergreen perennial shrub from the Lamiaceae mint family [1], which has been widely used for a long time as a food spice and folk medicine. More than 20 varieties of rosemary plants have been found worldwide so far. Rosemary contains a number of phytochemicals, including rosmarinic acid, carnosol, carnosic acid, oleanolic acid, ursolic acid (Figure 1). Those compounds and some of their derivatives like 3-*O*-acyl ursolic acid have been demonstrated to have multiple physiological activities such as antioxidant [2,3,4,5], antibacterial [2,3,6], anti-cancer [7,8,9], anti-diabetic [10], antithrombotic [11], and anti-HIV effects [12,13], and could be a therapeutic agent in neurodegenerative disease treatment [14,15,16]. Rosemary and its extracts are currently witnessing high demand in the global market due to the significant physiological activity. Therefore, the establishment of an analytical method for the rapid and accurate detection of the main chemical components is of great significance for the quality control of rosemary resources and for guiding its safe application in the food field.

However, to date, no work has appeared for the simultaneous determination of rosmarinic acid, carnosol, carnosic acid, oleanolic acid and ursolic acid from rosemary with high-performance liquid chromatography (HPLC). A few HPLC-ultraviolet detector methods have been reported for the simultaneous determination of two or three compounds that are similar in polarity from rosemary extracts [17,18], but the polarities of these five components are significantly different, and the diterpenes and triterpenoids in rosemary show weak ultraviolet absorbance. Simultaneous analysis of these five compounds with HPLC-ultraviolet detector showed great drift and significant baseline noise the impact of which on the precision and accuracy is unacceptable. Although HPLC or gas chromatography coupled with mass spectrometry is able to quantify these compounds [19,20], it has its own practical limitations including cost and is not commonly available in regular laboratories. Therefore, it is necessary to develop an analytical method to quantify the five functional ingredients to improve the resources comprehensive exploitation and utilization of rosemary.

Evaporative light scattering detection (ELSD) is a universal and non-specific mass detector, which associated with HPLC has gained popularity for both quantitative and qualitative analysis of non-volatile compounds that possess weak chromophores [21]. Meanwhile, such kind of detectors has been shown some greatest advantages including the sensitivity, stability and compatibility with gradient elution [22]. Some reports have been successfully applied for the determination of oleanolic acid and ursolic acid in different medicinal plants using HPLC-ELSD [23,24].

Thus, the aim of the present study was to develop and validate a rapid, simple, reliable HPLC-ELSD method to simultaneously determine rosmarinic acid, carnosol, carnosic acid, oleanolic acid and ursolic acid in rosemary. This method could be easily adapted to other functional ingredients analysis and used as a tool for rosemary quality control.

## 2. Results and Discussion

### 2.1. Selection of High-Performance Liquid Chromatography With Evaporative Light Scattering Detection (HPLC-ELSD) Conditions

**Figure 2:**
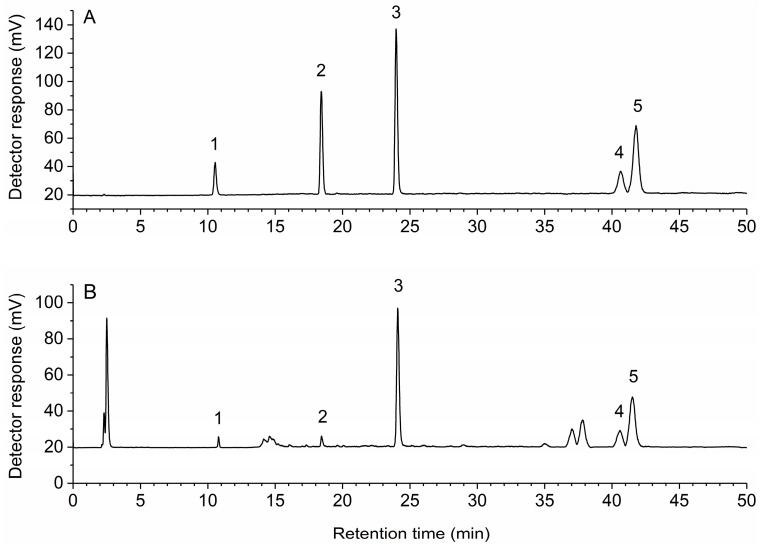
High-performance liquid chromatography with evaporative light scattering detection (HPLC-ELSD) chromatograms of multi-standards (**A**) and samples (**B**). 1, rosmarinic acid; 2, carnosol; 3, carnosic acid; 4, oleanolic acid; 5, ursolic acid.

To get the best separation efficiency, different kinds of solvent systems were tested and compared. Better resolution was recorded with methanol-0.6% acetic acid than acetonitrile-0.1% trifluoroacetic acid or methanol-0.1% trifluoroacetic acid. After repeated adjustment of the composition and elution time of the mobile phase, rosmarinic acid, carnosol, carnosic acid achieved a baseline separation, the maximum resolution of oleanolic acid and ursolic acid was 0.998. Different column temperatures of 20, 25, 30, 35 °C were optimized, and 30 °C was selected as preferable. The important parameter of ELSD conditions is the drift tube temperature which was evaluated at different drift tube temperatures from 60 to 80 °C. Good peak symmetries and efficiencies were achieved with a drift tube temperature at 70 °C. Thus, the optimal chromatographic conditions were as follows: flow rate, 1 mL/min; column temperature, 30 °C; sample injection volume, 20 μL; mobile phase, methanol (A) and 0.6% acetic acid (B) with a gradient elution of 40–50% A at 0–8 min, 50–83% A at 8–10 min, 83–85% A at 10–25 min, 85% A at 25–50 min. The representative HPLC-ELSD chromatograms of the multi-standards and samples are shown in Figure 2.

### 2.2. Calibration Curves, Limits of Detection and Quantification

The linearity studies of five reference compounds were performed using the developed aforementioned HPLC-ELSD methods. The calibration curves were calculated by double logarithmic transformation of the peak area and the concentration of the reference solution injected. The range and correlation coefficients are presented in Table 1. All calibration curves showed good linearity (R^2^ ≥ 0.991) within the test ranges. Limits of detection of the five analytes varied from 1.3 to 8.6 μg/mL and limits of quantification ranged from 4.3 to 28.8 μg/mL. These results demonstrated that the HPLC-ELSD method is sensitive and precise for the quantitative determination of five analytes in rosemary.

### 2.3. Precision, Repeatability, and Robustness

The intra-day and inter-day precision were investigated with the standard solution under the selected optimal conditions; the results are shown in Table 2. The relative standard deviation (RSD) values of intra-day and inter-day variations of the five analytes were all less than 3.1%. This indicated that the method developed by the instrument was in good precision condition. To determine repeatability, the same batch of rosemary samples was analyzed in six replicates as described earlier. The results displayed a maximum RSD at 3.8%. Robustness was tested with a sample solution at room temperature and analyzed at 0, 2, 4, 6, 8, 12 h after preparation, respectively. The RSD values among measurements ranged from 1.5 to 3.4% (Table 2), which indicated that rosemary extract remained stable at least for 12 h.

### 2.4. Accuracy

The accuracy of the developed method was evaluated by adding known amounts of reference standards to rosemary samples and comparing the extracted amounts to the added amounts. The overall recoveries of the five components were between 95.5% and 100.8% with RSD less than 3.1% (Table 2). Considering the results of the recovery test, the method was thus deemed precise and accurate.

### 2.5. Sample Analysis

The validated analytical method was applied for the analysis of rosmarinic acid, carnosol, carnosic acid, oleanolic acid and ursolic acid in rosemary from different locations in China. Each sample was extracted, processed, and quantified in accordance with the methods mentioned above and was determined in triplicate. Figure 2 depicts a representative chromatogram demonstrating the separation of the five analytes under the optimized conditions. As shown in Table 3, there were significant differences among the contents of the five analytes in different locations. Thus, the establishment of a method for simultaneous determination of bioactive compounds in rosemary is essential to improve the quality control of raw materials.

## 3. Materials and Methods

### 3.1. Reagents and Samples

All reagents and solvents used were at least of analytical grade. Rosmarinic acid (≥98%) and carnosol (≥98%), carnosic acid (≥97%), oleanolic acid (≥98%) and ursolic acid (≥98%) were obtained from Sigma-Aldrich (St. Louis, MO, USA). HPLC-grade methanol and acetonitrile were gained from Spectrum Chemical Mfg Corp (Gardena, CA, USA), acetic acid was of analytical grade and supplied from Sinopharm Chemical Reagent (Shanghai, China), HPLC grade trifluoroacetic acid was purchased from Sigma-Aldrich. Ultrapure water for chromatographic was prepared using a Milli-Q water purification system (Millipore, Billerica, MA, USA). Rosemary materials consisted of dried leaves were acquired from Hunan Sunfull Bio-tech (Changsha, China).

### 3.2. Preparation of Standards and Samples

The five reference standards were weighed accurately and dissolved in methanol in a volumetric flask (25 mL) to make a mixed stock solution, and the concentrations of each compound were: rosmarinic acid, 172.0 μg/mL; carnosol, 52.9 μg/mL; carnosic acid, 306.0 μg/mL; ursolic acid, 294.0 μg/mL; oleanolic acid, 396.0 μg/mL. The working standard solutions were prepared from the concentrated stock solution by further appropriate dilution in the methanol. All the solutions were filtered through a 0.45 μm nylon membrane and stored at 4 °C prior to analysis.

Rosemary materials were cut into pieces and sifted through 40 meshes. A 0.5 g sample was accurately weighed and put into a 100 mL volumetric flask, and then 100 mL of methanol was added, which was weighted again and recorded. Then using ultrasonic bath KQ-5200DE (Kun Shan Ultrasonic Instruments Co., Ltd., Jiangsu, China) at room temperature (25 ± 1 °C) for 40 min. Next, the sample was cooled to room temperature, and methanol was added to compensate for the loss of weight. This solution was filtered through a 0.45 μm membrane before analysis.

### 3.3. Instrumentation and HPLC-ELSD Conditions

An HPLC instrument consisted of SCL-10Avp (Shimadzu, Kyoto, Japan) chromatographic control system, two LC-10ATvp pumps (Shimadzu), a CTO-10ASvp column oven (Shimadzu), and a LC Solution chromatographic workstation. The system was equipped with a Zorbax SB-C18 column (4.6 mm × 250 mm, 5 μm, Agilent, Palo Alto, CA, USA). The mobile phase was methanol (A) and 0.6% acetic acid in water (B) with a gradient elution of 40–50% A at 0–8 min, 50–83% A at 8–10 min, 83–85% A at 10–25 min, 85% A at 25–50 min. During the elution program, the flow rate was 1 mL/min, the temperature of column oven was 30 °C, and the injection volume was 20 μL. SofTA Model 400 ELSD (SofTA Corporation, Boulder, CO, USA) was connected to the HPLC instrument in series mode. The drift tube temperature of ELSD was 70 °C, nebulizer temperature was 30 °C, and the nitrogen gas pressure was 40 Psi.

### 3.4. Calibration Curves, Limits of Detection and Quantification

For calibration, the above said mixed working solution with at least six different concentrations (performed in triplicate) were analyzed, and the linear calibration curves were calculated by plotting the logarithm of peak areas versus the logarithm of concentration injected for each analysis. The mixed solution of the five reference compounds was further diluted to a series of concentrations of methanol for the gain of the limit of detection (LOD) and limit of quantification (LOQ). The LOD and LOQ under the present chromatographic conditions were determined at the signal-to-noise ratios of each analyte of about 3 and 10, respectively.

### 3.5. Precision, Repeatability, and Robustness

The intra-day and inter-day variations, which were analyzed to determine the precision of the developed quantitative method, were investigated by determining the five analytes in six replicates within one day and by duplicating the experiments on three consecutive days. The RSD value of the peak area was adopted to evaluate precision. Repeatability was confirmed with six independent analytical sample solutions prepared according to the methods described above and variations expressed by RSD. To assess the robustness of the developed assay, the sample was stored at room temperature, and injected into the HPLC apparatus at 0, 2, 4, 6, 8, 12 h after preparation, respectively.

### 3.6. Accuracy

A recovery test was performed by adding known amounts of the five standards into the above sample separately to evaluate the accuracy of this method. The spiked samples were then extracted, processed, and quantified in accordance with the methods mentioned above. Six replicates were performed for the test. The average recovery percentage was calculated by the formula: recovery (%) = (observed amount − original amount)/spiked amount × 100%.

## 4. Conclusions

In this study, a simple, sensitive and reliable quantitative method for simultaneous analysis of rosmarinic acid, carnosol, carnosic acid, oleanolic acid and ursolic acid in rosemary was developed based on HPLC-ELSD. On the other hand, we have tried to improve the separation degree of oleanolic acid and ursolic acid by changing the flow rate, column temperature and adjusting the mobile phase ratio. However, the maximum resolution is still 0.998, which is also related to the fact that ELSD cannot be used as a mobile phase in the absence of volatile salts, which restricts the choice of mobile phase. It is possible that we can further improve the separation degree by changing the high performance column, adjusting the pH of the mobile phase, and adopting some other positive ways etc. Generally, the results for the validation parameters such as linearity, LOQ, LOD, precision, repeatability, robustness, and accuracy were acceptable. Compared to existing techniques, such as ultra-performance liquid chromatography or HPLC-mass spectrometry, the simplicity of the method allows it to be widely used in laboratories that lack sophisticated analytical instruments. The established method could be used for quality evaluation of rosemary and its extracts, and is of great significance for the quality control of rosemary resources and for guiding its safe application in the food field.

## Figures and Tables

**Table 1:** Calibration curves, limit of detection (LOD) and limit of quantification (LOQ) data of investigated analyte by HPLC-ELSD.

Analytes	Calibration Curves	R^2^	Linear Range (μg/mL)	LOD (μg/mL)	LOQ (μg/mL)
Rosmarinic acid	y = 1.4574x + 4.4181	0.998	17.2–172.0	6.7	20.5
Carnosol	y = 1.3384x + 5.7104	0.993	5.3‒52.9	1.3	4.3
Carnosic acid	y = 1.4906x + 5.4499	0.999	30.6‒306.0	2.5	8.5
Oleanolic acid	y = 1.1558x + 5.3079	0.996	29.4‒294.0	8.6	28.8
Ursolic acid	y = 1.3198x + 5.2916	0.991	39.6‒396.0	5.7	19.0

**Table 2:** Precision, repeatability, robustness, and accuracy of 5 analytes.

Analytes	Precision (Relative Standard Deviation, RSD, %)		Repeatability (RSD, %)	Robustness (RSD, %)	Accuracy (*n* = 6)	
	Intraday (*n* = 6)	Interday (*n* = 6)			Mean	RSD%
Rosmarinic acid	2.6	3.1	2.5	2.7	95.5	3.1
Carnosol	1.6	1.9	3.6	2.6	98.8	2.5
Carnosic acid	2.0	2.1	2.0	1.5	100.8	1.1
Oleanolic acid	1.2	1.4	3.0	2.9	97.1	2.6
Ursolic acid	2.2	2.1	3.8	3.4	99.3	3.0

**Table 3:** Contents of rosmarinic acid, carnosol, carnosic acid, oleanolic acid and ursolic acid in rosemary from different locations in China. (%, mean values ± standard deviation (SD), *n* = 3).

Origin	Rosmarinic Acid	Carnosol	Carnosic Acid	Oleanolic Acid	Ursolic Acid
Guizhou	5.22 ± 0.07	1.24 ± 0.05	2.51 ± 0.04	1.13 ± 0.03	1.02 ± 0.06
Guangxi	4.64 ± 0.09	0.53 ± 0.02	3.15 ± 0.03	1.55 ± 0.04	1.85 ± 0.03
Yunnan	1.31 ± 0.03	0.68 ± 0.02	2.83 ± 0.02	1.34 ± 0.02	2.75 ± 0.04
Hubei	2.34 ± 0.06	0.94 ± 0.02	2.44 ± 0.03	0.66 ± 0.03	2.36 ± 0.04
Guangdong	1.53 ± 0.07	0.46 ± 0.03	2.35 ± 0.04	1.15 ± 0.03	2.24 ± 0.06
